# Pathogenicity prediction for noncanonical splice-altering variants based on multimodal feature fusion

**DOI:** 10.1093/bib/bbag291

**Published:** 2026-06-04

**Authors:** Xiaoyan Li, Zhen Peng, Yiran Zhao, Shuhan Wang, Xingpeng Zhou, Xiongjian Luo, Yansen Su, Chunhou Zheng, Junfeng Xia

**Affiliations:** Information Materials and Intelligent Sensing Laboratory of Anhui Province and School of Life Sciences and Medical Engineering, Anhui University, No. 111 Jiulong Road, Hefei, Anhui, 230601, China; Key Laboratory of Computational Neuroscience and Brain-Inspired Intelligence (Fudan University), Ministry of Education, No. 220 Handan Road, Yangpu District, Shanghai, 200433, China; Institutes of Physical Science and Information Technology, Anhui University, No. 111 Jiulong Road, Hefei, Anhui, 230601, China; Information Materials and Intelligent Sensing Laboratory of Anhui Province and School of Life Sciences and Medical Engineering, Anhui University, No. 111 Jiulong Road, Hefei, Anhui, 230601, China; Information Materials and Intelligent Sensing Laboratory of Anhui Province and School of Life Sciences and Medical Engineering, Anhui University, No. 111 Jiulong Road, Hefei, Anhui, 230601, China; Information Materials and Intelligent Sensing Laboratory of Anhui Province and School of Life Sciences and Medical Engineering, Anhui University, No. 111 Jiulong Road, Hefei, Anhui, 230601, China; Department of Psychiatry and Psychosomatics, Zhongda Hospital, School of Medicine, Advanced Institute for Life and Health, Jiangsu Provincial Key Laboratory of Brain Science and Medicine, Southeast University, No. 87 Dingjiaqiao Road, Nanjing, Jiangsu, 210009, China; School of Artificial Intelligence, Anhui University, No. 111 Jiulong Road, Hefei, Anhui, 230601, China; School of Artificial Intelligence, Anhui University, No. 111 Jiulong Road, Hefei, Anhui, 230601, China; Information Materials and Intelligent Sensing Laboratory of Anhui Province and School of Life Sciences and Medical Engineering, Anhui University, No. 111 Jiulong Road, Hefei, Anhui, 230601, China

**Keywords:** pathogenicity prediction, splicing variants, multimodal fusion, model interpretability, transformer

## Abstract

Splice-altering variants (SAVs) are the second most prevalent class of pathogenic genetic variants and are strongly associated with the occurrence and development of various diseases. However, current computational tools exhibit limited predictive capability beyond canonical GT-AG splice sites, making accurate assessment of noncanonical SAV pathogenicity a considerable challenge. To address this limitation, we developed MOSAIC (multimodal feature fusion for noncanonical splice-altering variants pathogenicity prediction), a deep learning framework designed for precise assessment of noncanonical SAV pathogenicity. MOSAIC integrates long-range contextual signals derived from a pretrained DNA language model, local sequence features captured from multi-scale convolutional neural networks, and functional annotations. By employing a transformer encoder and a gated fusion module, the model adaptively integrates these multimodal features. Benchmarking across multiple independent datasets demonstrated that MOSAIC consistently outperforms existing state-of-the-art methods, such as CADD and SpliceAI. It remains highly accurate and robust when evaluated on rare variants, gene-independent contexts, and the largest subset where all comparative methods yielded outputs. Furthermore, feature importance analysis revealed that long-range dependencies in DNA sequences and transformer-based integration were critical contributors to model performance. Interpretability analyses indicated that MOSAIC could identify key regulatory sequence motifs associated with transcription factors and RNA-binding proteins, offering mechanistic insight into how noncanonical SAVs disrupt splicing regulation and contribute to pathogenic processes. Overall, MOSAIC offers an accurate and interpretable framework for predicting the pathogenicity of noncanonical SAVs, thereby serving as a dependable computational tool for genetic diagnostics and precision medicine applications. MOSAIC source code and data are available at https://github.com/Lilab-genomics/MOSAIC.

## Introduction

Advances in next-generation sequencing (NGS) have markedly improved the diagnosis of Mendelian diseases, with diagnostic yields rising from 16%–25% in early studies [1–3] to 35%–60% in recent cohorts [4, 5]. Nevertheless, a substantial proportion of cases remain genetically unexplained [6]. A major challenge is the interpretation of splice-altering variants (SAVs), particularly noncanonical variants located outside the highly conserved ±1/±2 splice-site dinucleotides [7]. Aberrant splicing is estimated to underlie 15%–50% of disease-associated variants [8–12], yet the functional consequences of SAVs are often difficult to infer from sequence context alone. Although American College of Medical Genetics and Genomics (ACMG) guidelines consider canonical splice-site variants strong evidence of pathogenicity in genes with loss-of-function mechanisms [13], noncanonical SAVs remain much harder to interpret because of weaker sequence conservation and limited expert-curated annotations [14, 15]. Emerging evidence suggests that noncanonical SAVs may represent a prevalent yet underrecognized cause of Mendelian diseases [15, 16]. Therefore, enhancing the accuracy of pathogenicity prediction for noncanonical SAVs is essential for elucidating the genetic basis of unresolved cases and clinical decision-making. Moreover, the interpretability of predictive models is crucial to ensure their integration into genomics workflows and to support transparent variant classification.

Although canonical SAVs are readily identified using current computational approaches, noncanonical SAVs remain significantly more difficult to interpret. In recent years, computational methods ranging from traditional machine learning to deep learning have been widely applied to assess SAV pathogenicity. Established computational methods can be broadly categorized into general-purpose pathogenicity tools and SAV-specific tools. General-purpose tools, such as CADD [17], SIGMA [18], PhD-SNPg [19], ML-GVP [20], VARITY [21], and EVE [22], are designed to evaluate the pathogenicity of diverse variant types across the genome by integrating diverse variant features. However, this approach lacks precision in accurately predicting the pathogenicity of SAVs. To more directly and effectively assess SAV pathogenicity, several SAV-specific pathogenicity prediction tools have been developed, including S-CAP [23], SQUIRLS [24], PDIVAS [25], MMSplice [26], SpliceAI [27], Pangolin [28], and DeltaSplice [29]. Among these, some methods have been developed specifically based on the molecular effects of splicing (i.e. the identification of SAVs). For example, SpliceAI [27] uses deep convolutional networks to model splicing signals from long genomic sequences, whereas Pangolin [28] further incorporates tissue-specific transcriptomic context. Others improve prediction by integrating multilevel biological features. For instance, S-CAP [23] applies region-specific gradient-boosted trees, and SQUIRLS [24] combines interpretable feature engineering with machine learning to support prediction of noncanonical SAV pathogenicity.

Despite these advances, predicting the pathogenicity of noncanonical SAVs remains challenging. Progress has been limited by their complex, context-dependent regulatory mechanisms and the scarcity of high-quality validated datasets. Current limitations mainly involve feature extraction, model architecture, and interpretability. Because the mechanisms of noncanonical SAVs are not fully understood, expert-driven features often fail to capture complex regulatory patterns. Most existing methods also rely on single-source or single-level sequence information, limiting their ability to model complex variants. In addition, model interpretation is largely based on known biological knowledge, which restricts generalizability and the discovery of novel splicing mechanisms. Together, these limitations reduce predictive accuracy and hinder the clinical application of current tools.

To address these challenges, we developed MOSAIC, a multimodal framework for predicting the pathogenicity of noncanonical SAVs. By integrating long-range sequence context, local sequence features, and functional annotations, MOSAIC captures complex splicing regulatory signals through adaptive feature fusion. Across multiple benchmark datasets, MOSAIC consistently outperformed six existing methods and showed robust performance on rare variants. Interpretability and enrichment analyses further demonstrated that MOSAIC learned biologically meaningful features and accurately identified pathogenic variants enriched in regulatory regions and RNA-binding protein binding sites. These results highlight the potential of MOSAIC to improve noncanonical SAV interpretation in genetic diagnosis and precision medicine.

## Methods

### Data preparation

Single-nucleotide variants (SNVs) based on the GRCh38/hg38 assembly were collected from HGMD Professional (v2023.03) and ClinVar (release 1 December 2024). After high-confidence pathogenic and benign variants were selected and filtered for noncanonical splice-related categories, a genome position-based nearest-neighbor matching strategy was used to construct a balanced dataset. The matched dataset was split into training, validation, and independent testing sets, with the independent test set denoted as HDTesting. In addition, three supplementary evaluation datasets were generated: HDSTesting, composed of unmatched pathogenic variants and randomly selected benign controls; VarRareTest, containing rare variants with allele frequency < 0.01; and HDGTesting, a gene-separated test set used to assess cross-gene generalizability. Detailed filtering criteria and dataset construction procedures are provided in the Supplementary Methods.

### Multimodal feature extraction in MOSAIC

To systematically capture the complex regulatory patterns underlying noncanonical SAVs, three categories of complementary features were extracted for each input variant: DNA2vec features, GPN-MSA features, and functional annotation features. These feature types were designed to encode local sequence patterns, long-range contextual dependencies, and functional regulatory information, respectively.

#### DNA2vec features

The DNA2vec model [30] provides a framework for generating consistent vector embeddings of variable-length k-mers from DNA sequences, thereby overcoming limitations of traditional one-hot encoding. One-hot encodings scale poorly with increasing k-mer length and fail to capture semantic similarity, as all vectors remain equidistant. Inspired by the word2vec model used in natural language processing, DNA2vec employs a shallow two-layer neural network to learn dense representations of k-mers (typically 3–8 nucleotides in length). These k-mers are mapped into a low-dimensional embedding space where geometric proximity reflects biological or functional similarity.

The embeddings generated by DNA2vec can be used to generate distributed representations for sequence fragments, identify neighborhoods of similar k-mers, or perform nucleotide concatenation analogy operations. For each variant, a DNA sequence fragment of length l is encoded as an l × d matrix, where d denotes the dimensionality of each k-mer embedding. This representation implicitly captures local sequence information of the variants. To reduce computational complexity, the high-dimensional DNA2vec embeddings were projected into a 64-dimensional space using principal component analysis (PCA), facilitating efficient downstream processing.

#### GPN-MSA features

GPN-MSA [31] is a DNA language model trained on multi-species whole-genome multiple sequence alignments (MSAs) and is designed to capture evolutionary conservation information within sequences. By leveraging large-scale, unsupervised training on whole-genome alignment data across multiple species, the model learns nucleotide-level conservation patterns and functional constraints, thereby generating comprehensive long-range contextual representations. Compared with traditional k-mer-based encodings or single-species models, this approach more effectively utilizes evolutionary information. Using this model, each DNA sequence fragment of length $l$ is encoded into a 768-dimensional representation. To mitigate redundancy and computational burden, PCA was employed to reduce the dimensionality of these embeddings to 512 dimensions. This approach retains the primary evolutionary signals while enhancing computational efficiency. Centered on the variant position, DNA sequence fragments of 64 bp in length were selected as model inputs. We directly used the pretrained GPN-MSA model to generate embeddings, and no additional preprocessing or quality-control filtering was applied to the input MSAs prior to embedding. A comparative analysis across various input lengths was performed on the validation set using a multilayer perceptron (Supplementary Table S1).

#### Functional annotation features

Functional annotation features (totaling 47 dimensions) were used to capture the biological context of each noncanonical SAV, including splicing-specific features, sequence and genomic structure features, conservation scores, and epigenetic features. Details of functional annotation feature construction are provided in the Supplementary Methods, and the full feature list is provided in Supplementary Table S2. Collectively, these features characterize the splicing regulatory potential, genomic context, evolutionary constraints, and chromatin state of variants within their local genomic environment.

### MOSAIC framework

**Figure 1:**
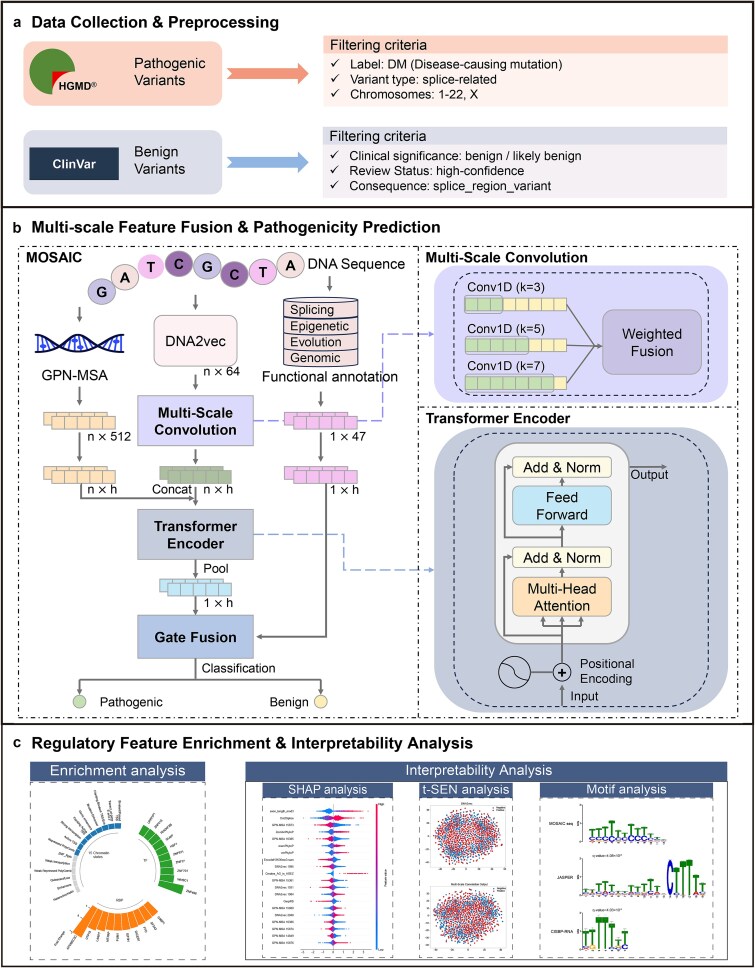
Overview of the MOSAIC architecture. The framework comprises three main modules: (a) data collection and preprocessing module; (b) multimodal feature fusion and pathogenicity prediction module; and (c) regulatory feature enrichment and interpretability analysis module.

An overview of the MOSAIC architecture is shown in Fig. 1. The framework consists of three core components: (i) data collection and preprocessing module, (ii) multimodal feature fusion and pathogenicity prediction module, and (iii) regulatory feature-based enrichment and interpretability analysis module. Module (i) systematically screens and harmonizes noncanonical SAVs using curated data from the HGMD [32] and ClinVar [33] databases, yielding a high-quality training dataset. Module (ii) extracts multimodal features for each variant: DNA2vec features, GPN-MSA features, and functional annotation features. DNA2vec embeddings derived from the input sequence are first processed by a multi-scale convolutional neural network to capture local sequence patterns at different receptive fields. The resulting representations are then concatenated with GPN-MSA features and passed to a Transformer encoder to model long-range contextual dependencies, yielding a unified sequence representation. Functional annotation features are subsequently projected into the same embedding space and adaptively integrated with the sequence representation through a gated fusion module, producing a unified feature vector for pathogenicity prediction. Module (iii) performs an enrichment analysis based on regulatory features to evaluate the reliability of the model predictions. In addition, interpretability analyses, such as feature importance assessment and sequence motif analysis, are performed to elucidate the predictive basis and decision-making mechanisms of the model from multiple perspectives. These analyses collectively enhance the biological interpretability and transparency of the framework.

### Encoding local sequence patterns via a multi-scale convolution module

Local sequence patterns are extracted from DNA2vec features using a multi-scale convolution module. Let the input feature tensor be denoted as follows:


$$ X\in{R}^{B\times L\times{C}_{in}}, $$

where $B$ denotes the batch size, $L$ denotes the sequence length, and ${C}_{in}$ denotes the number of input channels.

First, a linear projection is applied to map the input features to a unified channel dimension ${C}_{out}$:


(1) \begin{equation*} {X}^{\prime }= Linear(X),\kern0.5em {\mathrm{X}}^{\prime}\in{\mathrm{R}}^{\mathrm{B}\times \mathrm{L}\times{\mathrm{C}}_{\mathrm{out}}}. \end{equation*}

The projected features are then transposed into the format required for one-dimensional convolution:


$$ {X}^{\prime \prime}\in{R}^{B\times{C}_{out}\times L}. $$

Multiple one-dimensional convolutional branches with different kernel sizes ${s}_i\in \left\{3,5,7\right\}$ are applied in parallel to extract local features at multiple scales. A performance comparison of multi-scale convolution with varying kernel sizes on the validation set is provided in Supplementary Table S3.


(2) \begin{equation*} {\displaystyle \begin{array}{c}{F}_i= Conv1{D}_{s_i}\left({X}^{\prime \prime}\right),\kern0.5em i=1,\dots, K,\kern0.5em K=3.\end{array}} \end{equation*}

All convolutions employ a same padding strategy to preserve the sequence length dimension across branches.

To model inter-channel dependencies and enhance feature discrimination, each convolutional branch is equipped with a Squeeze-and-Excitation (SE) mechanism. This module performs global average pooling followed by a gating operation to adaptively recalibrate channel-wise feature responses:


(3) \begin{equation*} {\displaystyle \begin{array}{c}{F_i}^{SE}={F}_i\odot SE\left({F}_i\right),\#\end{array}} \end{equation*}

where ⊙ denotes element-wise multiplication with channel-wise broadcasting.

Subsequently, features from different convolutional branches are integrated using a learnable weighted fusion mechanism. First, a trainable weight vector


$$ w=\left[{w}_1,{w}_2,\cdots, {w}_K\right], $$

is normalized using the softmax function:


(4) \begin{equation*} {\displaystyle \begin{array}{c}{\alpha}_i=\frac{\mathit{\exp}\left({w}_i\right)}{\sum_{j=1}^K\mathit{\exp}\left({w}_j\right)}.\end{array}} \end{equation*}

Finally, the fused local sequence representation is obtained as:


(5) \begin{equation*} {\displaystyle \begin{array}{c}{F}_{local}=\sum_{i=1}^K{\alpha}_i{F_i}^{SE}.\end{array}} \end{equation*}

### Learning DNA sequence representations using a transformer encoder module

To jointly capture local sequence features and long-range contextual dependencies, MOSAIC concatenates the local representations generated by the multi-scale convolution module


$$ {F}_{local}\in{R}^{B\times L\times{d}_{model}}, $$

with GPN-MSA features


$$ {F}_{pre}\in{R}^{B\times L\times{d}_{model}}, $$

along the feature dimension to obtain a unified sequence representation:


(6) \begin{equation*} {\displaystyle \begin{array}{c}{F}_{concat}=\left[{F}_{local},{F}_{pre}\right]\in{R}^{B\times L\times 2{d}_{model}}.\end{array}} \end{equation*}

To incorporate positional information, sinusoidal positional encodings


$$ PE\in{R}^{L\times 2{d}_{model}}, $$

are added to the concatenated features. These encodings are defined as: 


(7) \begin{align*} {PE}_{\left( pos,2i\right)}&=\mathit{\sin}\left(\frac{pos}{10000^{2i\!\left/ \!2{d}_{model}\right.}}\right),\nonumber \\& {PE}_{\left( pos,2i+1\right)} =\mathit{\cos}\left(\frac{pos}{10000^{2i\!\left/ \!2{d}_{model}\right.}}\right). \end{align*}

where $pos$ denotes the sequence position and $i$ denotes the feature dimension index. The positional encodings are broadcast along the batch dimension. The final input representation to the encoder is given by:


(8) \begin{equation*} {\displaystyle \begin{array}{c}{X}_0={F}_{concat}+ PE.\end{array}} \end{equation*}

The encoder consists of three stacked transformer encoder layers. A performance comparison with different numbers of transformer encoder layers on the validation set is provided in Supplementary Table S4. For the $l$-th layer, the input representation is


$$ {X}_{l-1}\in{R}^{B\times L\times 2{d}_{model}}. $$

The multi-head self-attention (MHSA) mechanism is defined as:


(9) \begin{equation*} {\displaystyle \begin{array}{c} Attention\left(Q,K,V\right)= Softmax\left(\frac{QK^T}{\sqrt{d_k}}\right)V.\end{array}} \end{equation*}

The query, key, and value matrices are computed as:


(10) \begin{equation*} {\displaystyle \begin{array}{c}Q={X}_{l-1}{W}^Q,\kern0.5em K={X}_{l-1}{W}^K,\kern0.5em V={X}_{l-1}{W}^V.\end{array}} \end{equation*}

The position-wise feed-forward network (FFN) is defined as:


(11) \begin{equation*} {\displaystyle \begin{array}{c} FFN(x)= GELU\left(x{W}_1+{b}_1\right){W}_2+{b}_2.\end{array}} \end{equation*}

Each encoder layer adopts residual connections followed by layer normalization:


$$ {X}_l^{\prime }= LayerNorm\left({X}_{l-1}+ MHSA\left({X}_{l-1}\right)\right), $$

(12) \begin{equation*} {\displaystyle \begin{array}{c}{X}_l= LayerNorm\left({X}_l^{\prime }+ FFN\left({X}_l^{\prime}\right)\right),\kern0.5em l=1,\dots, 3.\end{array}} \end{equation*}

After the final encoder layer, a sequence representation that integrates both local and global information is obtained:


(13) \begin{equation*} {\displaystyle \begin{array}{c}{X}_{out}\in{R}^{B\times L\times 2{d}_{model}}.\end{array}} \end{equation*}

To derive a fixed-length sequence-level representation, an attention pooling mechanism is applied to aggregate the sequence features:


(14) \begin{equation*} {\displaystyle \begin{array}{c}{x}_{seq}=\sum_{i=1}^L{\beta}_i{X}_{out}^{(i)},\kern0.5em \beta = Softmax\left({X}_{out}{W}_a\kern0em \right)\end{array}} \end{equation*}

Here, the softmax operation is applied along the sequence length dimension, and ${\beta}_i$ denotes the attention weight corresponding to the $i$-th position in the sequence.

### Adaptive integration of sequence representations and functional annotations via a gated fusion module

To effectively integrate sequence representations with functional annotation features, MOSAIC employs a lightweight gated fusion module (Supplementary Fig. S1). The sequence-level representation is denoted as:


$$ {x}_{seq}\in{R}^{2{d}_{model}}, $$

and the functional annotation features are denoted as:


$$ {x}_{bio}\in{R}^{2{d}_{model}}. $$

These two feature types are concatenated along the feature dimension and passed through a fully connected layer to generate a gating vector:


(15) \begin{equation*} {\displaystyle \begin{array}{c}g=\sigma \left(\left[{x}_{seq};{x}_{bio}\kern0.00em \right]{W}_g+{b}_g\right),\kern0.5em {W}_g\in{R}^{4{d}_{model}\times 2{d}_{model}}.\end{array}} \end{equation*}

where $\sigma$ denotes the sigmoid activation function, and


$$ g\in{\left[0,1\right]}^{2{d}_{model}} $$

is used to adaptively control the fusion proportion between the two feature types.

The final integrated feature vector is computed as:


(16) \begin{equation*} {\displaystyle \begin{array}{c}{x}_{fused}=g\odot{x}_{seq}+\left(1-g\right)\odot{x}_{bio}.\end{array}} \end{equation*}

### Prediction module

The fused feature representation ${x}_{fused}$ is fed into a fully connected prediction head to perform binary classification. The process begins with layer normalization followed by a nonlinear mapping:


(17) \begin{equation*} {\displaystyle \begin{array}{c}h= GELU\left( Linear\left( LayerNorm\left({x}_{fused}\right)\right)\right).\end{array}} \end{equation*}

Subsequently, dropout regularization and a linear transformation are applied. The predicted probability is then obtained using a sigmoid function:


(18) \begin{equation*} {\displaystyle \begin{array}{c}\hat{y}=\sigma \left( Linear\left( Dropout(h)\right)\right).\end{array}} \end{equation*}

where $\hat{y}\in \left[0,1\right]$, and larger values indicate a higher probability that the variant is pathogenic.

### Model training

MOSAIC is trained using the Adam optimizer [34] with the initial learning rate set to ${\eta}_0=1\times{10}^{-4}$. A weight decay coefficient ($\lambda =1\times{10}^{-3}$) is applied for regularization to mitigate the risk of overfitting. The model is configured with hidden dimensionality (${d}_{model}=256$), and the transformer encoder consists of three layers. The training data are partitioned into training and validation sets at a ratio of 80% to 20%. To improve training stability and prevent overfitting, an early stopping mechanism is employed during training. Training is automatically terminated if the validation AUC does not improve for $p=5$ consecutive epochs. The stopping criterion is defined as:


(19) \begin{equation*} {\displaystyle \begin{array}{c} Stop\ if\ {AUC}_{val}^{(t)}\le{\mathit{\max}}_{k\in \left\{t-p,\dots, t-1\right\}}{AUC}_{val}^{(k)},\kern0.5em t\ge p+1.\end{array}} \end{equation*}

where ${AUC}_{val}^{(t)}$ denotes the AUC value on the validation set at the $t$-th epoch. To further enhance the generalizability of the model, dropout regularization is introduced during training by randomly deactivating a subset of neurons, thereby reducing reliance on local features. The dropout rate is set to 0.2.

Based on validation performance, an adaptive learning rate scheduling strategy is employed. When the validation AUC fails to improve for $q=2$ consecutive epochs, the learning rate is halved. A minimum learning rate threshold is imposed at ${\eta}_{min}=1\times{10}^{-6}$, and the update rule is defined as:


(20) \begin{equation*} {\displaystyle \begin{array}{c}\ {\eta}^{\left(t+1\right)}=\mathit{\max}\left(\frac{\eta (t)}{2},{\eta}_{min}\right).\kern0.5em if\ {AUC}_{val}^{(t)}\le{\mathit{\max}}_{k\in \left\{t-q,\dots, t-1\right\}}{AUC}_{val}^{(k)}.\end{array}} \end{equation*}

Training is conducted using a fixed batch size of 256. Both AUC and AUPRC are used as performance evaluation metrics during training and validation. The model parameters achieving the highest validation AUC are selected for final deployment. The training and validation loss curves are provided in Supplementary Fig. S2 to illustrate the training dynamics. The test set was strictly held out for final performance evaluation only and was not used at any stage of model training, architecture selection, or hyperparameter tuning.

### Model performance evaluation

To evaluate the predictive performance of MOSAIC, we conducted comparative analyses against six widely used tools for noncanonical SAV prediction, including CADD [17], MMSplice [26], SpliceAI [27], Pangolin [28], DeltaSplice [29], and SQUIRLS [24]. Direct retraining under a unified data split was not feasible given substantial differences in model design, input representation, and training pipeline. For tools that provide continuous scores or pathogenicity probabilities, thresholds recommended by the original authors were adopted to classify variants as pathogenic or benign. For tools lacking predefined thresholds, a range of thresholds was evaluated on the HGMD-Training dataset, and the threshold yielding the highest F1 score was selected as the optimal cutoff. For MOSAIC, a fixed threshold of 0.5 was applied.

During benchmarking, certain tools failed to produce predictions for a subset of variants due to the missing scores. To maintain consistent comparisons across models, missing predictions were imputed with a default value of zero. In contrast, MOSAIC successfully produced valid predictions for all input variants, achieving complete coverage.

Given the class distribution differences between pathogenic and benign variants, multiple evaluation metrics were employed to ensure robust performance assessment. Receiver operating characteristic (ROC) curves were plotted, and the area under the ROC curve (AUC) was computed to assess model discrimination performance on balanced datasets. In addition, precision-recall (PR) curves were generated, and the area under the PR curve (AUPR) was calculated to evaluate model performance on imbalanced datasets. Furthermore, threshold-dependent metrics, including accuracy (ACC), specificity, precision, recall, F1-score, and Matthews correlation coefficient (MCC), were also calculated to provide a comprehensive evaluation of classification performance. All ROC and PR curves, along with corresponding AUC and AUPR scores, were generated using the torchmetrics [35] package to ensure consistency and reproducibility of the results.

### Interpretability analysis

#### Feature importance analysis

Quantifying input feature importance in deep learning models remains a major challenge. To evaluate feature importance and mitigate the black-box nature of the model, Shapley Additive exPlanations (SHAP) [36] were employed. Rooted in cooperative game theory, SHAP quantifies feature importance by computing the average marginal contribution of each input feature across all possible feature combinations, providing a theoretically grounded and consistent framework for model interpretation. Specifically, DNA2vec, GPN-MSA, and functional annotation features were integrated into a unified input vector and fed through the MOSAIC model. SHAP values were approximated using the GradientShap method implemented in the Captum [37] interpretability library. The resulting feature attribution scores were visualized and analyzed to interpret the decision-making process underlying pathogenicity predictions.

#### Feature visualization analysis

To assess whether MOSAIC learns discriminative feature representations, 2D visualizations were generated using t-distributed stochastic neighbor embedding (t-SNE) [38]. By preserving local similarities in high-dimensional space, t-SNE enables clearer cluster separation of samples with different pathogenicity labels in low-dimensional projections. This property provides an intuitive illustration of the discriminative power of the learned representations. Specifically, DNA2vec, GPN-MSA, and functional annotation features were individually projected into a 2D space using t-SNE to examine the baseline distributions of the original input features across different pathogenicity classes. Subsequently, feature representations were extracted from multiple intermediate layers of the model, including the multi-scale convolution module, transformer encoder module, and gated fusion module. These intermediate and integrated representations were then visualized using t-SNE. By comparing the projections obtained at different layers, the clustering separation and discriminative capability of the learned representations after model training can be intuitively assessed.

#### DNA sequence motif analysis

Regulatory DNA motifs, which are short sequence elements playing functional roles in gene expression, frequently demonstrate clustered distributions within the genome. Their spatial organization is intricately associated with the precision of splicing regulation [39]. Previous studies have shown that noncanonical SAVs can induce aberrant splicing by disrupting these functional motifs, thereby contributing to pathogenic outcomes [40]. Therefore, systematically identifying high-weight motifs associated with noncanonical SAVs represents a key step toward elucidating their pathogenic mechanisms.

Based on this rationale, we performed motif analysis on DNA sequence features associated with noncanonical SAVs that were captured by the model. This analysis aimed to localize potential regulatory elements and dissect their pathogenic pathways. Specifically, positional attention weights from the MOSAIC attention pooling layer were first extracted, and regions with the highest weights were mapped back to the original DNA sequences to define candidate regulatory fragments. These sequences were analyzed using the MEME tool [41] to discover enriched motifs ranging from 10 to 15 bp in length, retaining the top 10 most statistically significant signals. Identified motifs were converted into position weight matrices (PWMs) and compared using the TOMTOM tool [42] against known transcription factors (TFs) DNA-binding motifs from the JASPAR database [43] and reported human RBP binding motifs from the CISBP-RNA database [44]. Statistical significance of motif matches was assessed following false discovery rate correction, with a q-value threshold of ≤0.05 used to define significant alignments. This analysis directly links sequence features learned by the model to experimentally validated regulatory elements, providing mechanistic insight into the pathogenic potential of noncanonical SAVs.

### Regulatory feature enrichment analysis of noncanonical SAVs

To investigate whether predicted pathogenic noncanonical SAVs preferentially localize to regulatory elements, we performed enrichment analysis using genome-wide candidate noncanonical SAVs annotated from CADD [17] and scored by MOSAIC. Variants predicted as pathogenic were tested for enrichment in chromatin states, TF-binding sites and RNA-binding protein (RBP)-binding regions using GREGOR [45], with matched control variants used as background. Detailed annotation procedures, data sources, and enrichment analysis settings are described in the **Supplementary Methods**.

## Results

### Overview of MOSAIC

The MOSAIC model processes each variant through three sequential stages (Fig. 1). First, for each input sequence, three complementary feature categories are extracted: GPN-MSA features, DNA2vec features, and functional annotation features. These feature categories are designed to represent long-range contextual dependencies, local sequence patterns, and functional regulatory information, respectively. Next, the DNA2vec features are fed into a multi-scale convolution module, which models local sequence patterns in parallel across multiple receptive fields and integrates to produce a unified local representation. This local sequence representation is concatenated with GPN-MSA features and jointly input into a multilayer transformer encoder, where long-range sequence dependencies are captured via an MHSA mechanism. Finally, this global sequence representation is adaptively integrated with functional annotation features through a gated fusion module to produce a final fused representation. This fused representation is subsequently fed into a fully connected classifier to perform binary classification, assigning each variant a label of 0 for benign or 1 for pathogenic. This design enables MOSAIC to effectively integrate complementary sequence and functional information for pathogenicity prediction.

### MOSAIC outperforms existing prediction tools on benchmark datasets

**Figure 2:**
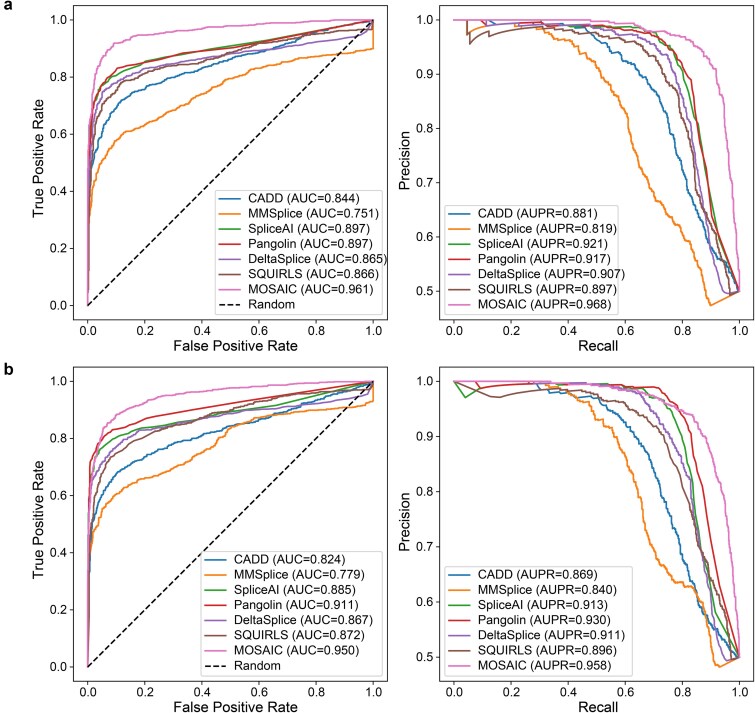
MOSAIC outperforms existing prediction models on the HDTesting and HDSTesting datasets. Precision-recall curves and receiver operating characteristic curves are shown for all models on the (a) HDTesting and (b) HDSTesting datasets.

We evaluated the performance of MOSAIC for the prediction of noncanonical SAVs and compared it with six established methods, including CADD [17], MMSplice [26], SpliceAI [27], SQUIRLS [24], Pangolin [28], and DeltaSplice [29]. On the HDTesting dataset, MOSAIC achieved superior performance, with an AUC of 0.961 and AUPR of 0.968, exceeding the AUC range of 0.751–0.897 and AUPR range of 0.819–0.921 observed for the other tools (Fig. 2a and Supplementary Table S5). Similarly, on the HDSTesting dataset, MOSAIC achieved an AUC of 0.950 and AUPR of 0.958. Among the remaining methods, Pangolin, SpliceAI, and SQUIRLS ranked next in terms of AUC, whereas Pangolin, SpliceAI, and DeltaSplice ranked next in terms of AUPR (Fig. 2b and Supplementary Table S6).

**Figure 3:**
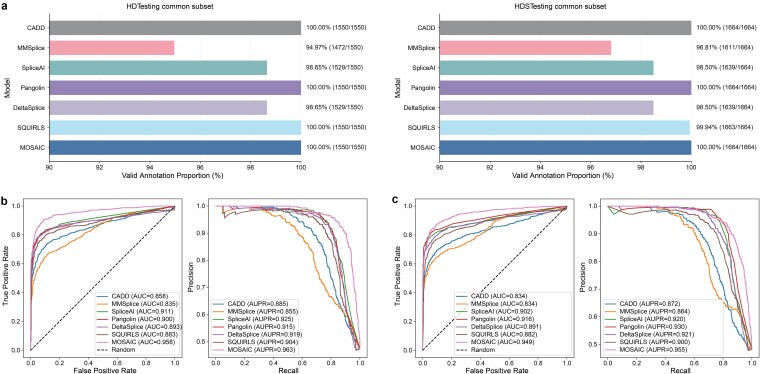
Model performance on common variant subsets. (a) Bar chart showing the number of variants with missing predictions for each model in the HDTesting and HDSTesting common subset. Precision-recall curves and receiver operating characteristic curves are shown for all models in HDTesting (b) and HDSTesting (c) common subsets.

We summarized the missing values in all predicted tools and found that some tools (i.e. MMSplice, SpliceAI, and DeltaSplice) had less than 6% missing values across the whole genome (Fig. 3a and Supplementary Tables S7 and S8). In contrast, MOSAIC, CADD, and SQUIRLS generated predictions for all variants across both datasets. To address missing predictions from some tools, performance was evaluated on the common subset of both datasets after excluding variants with missing annotations. The HDTesting common subset consisted of 689 pathogenic variants and 765 benign variants, while the HDSTesting common subset consisted of 766 pathogenic variants and 820 benign variants. On these filtered sets, MOSAIC consistently maintained the best performance, yielding AUC and AUPR values of 0.958 and 0.963 on the HDTesting common subset (Fig. 3b and Supplementary Table S9), and 0.949 and 0.955 on the HDSTesting common subset, respectively (Fig. 3c and Supplementary Table S10). Additionally, to further assess the robustness of MOSAIC, we performed five-fold cross-validation on the balanced dataset constructed using the close-by strategy. Cross-validation was conducted at the level of matched variant pairs to preserve the close-by matching design. The results demonstrated consistent and stable performance across the five-folds, with small standard deviations for all evaluation metrics (Supplementary Table S11). Together, these results support the robustness and stability of MOSAIC.

### MOSAIC enhances pathogenicity prediction for rare variants

**Figure 4:**
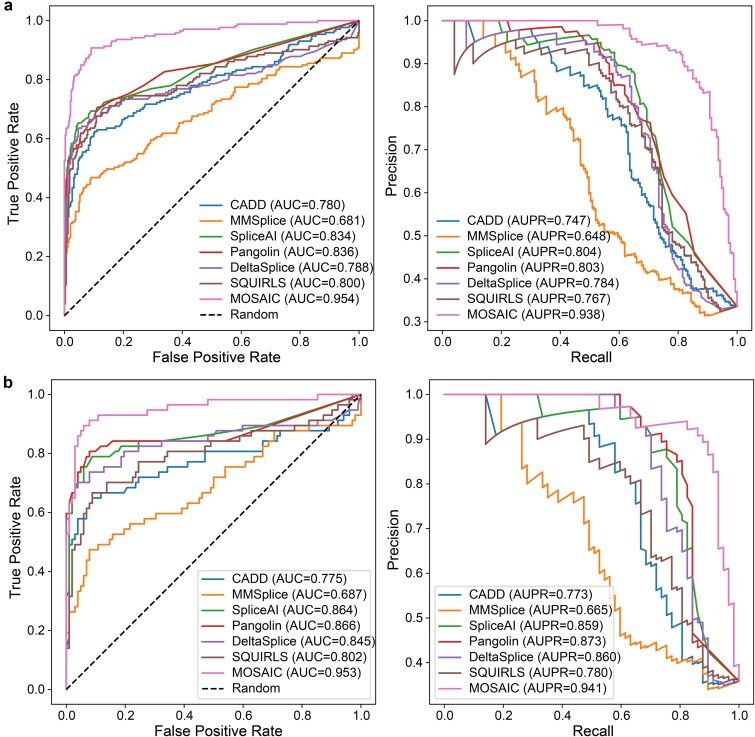
Model performance on rare variant and gene-independent tasks. (a) Receiver operating characteristic curves and precision-recall curves of MOSAIC and six comparison tools on the VarRareTest dataset. (b) Receiver operating characteristic curves and precision-recall curves of MOSAIC and six comparison tools on the HDGTesting dataset.

To evaluate MOSAIC performance on rare variant classification, the VarRareTest dataset comprising 515 variants with allele frequencies below 0.01 was used. MOSAIC achieved the highest predictive accuracy among all seven tools, with an AUC of 0.954 and AUPR of 0.938 (Fig. 4a). SpliceAI and Pangolin ranked next in performance. These results demonstrate that MOSAIC delivers superior pathogenicity prediction for rare variants compared to existing approaches, thereby offering a significant advantage.

### MOSAIC exhibits superior performance in gene-independent pathogenicity prediction

Performance under gene-independent conditions was evaluated using the HDGTesting dataset, which comprises 159 variants derived exclusively from genes absent from the training dataset. This design eliminated gene overlap between training and evaluation sets, preventing gene-level label leakage and enabling a stringent assessment of generalization. Comparative analysis against the other six prediction tools showed that MOSAIC achieved the highest performance, with an AUC of 0.953 and AUPR of 0.941 (Fig. 4b). Among the remaining methods, Pangolin, SpliceAI, and DeltaSplice ranked next in terms of AUC, whereas Pangolin, DeltaSplice, and SpliceAI ranked next in terms of AUPR (Fig. 4b). These results indicate that MOSAIC retained robust predictive performance in a gene-independent evaluation setting.

### Evaluation of contributions of MOSAIC features and model components via ablation analysis

**Figure 5:**
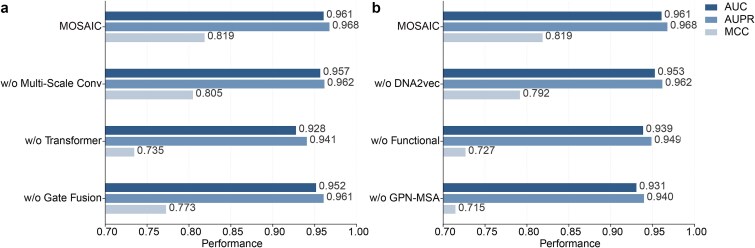
Ablation experiment results. Performance of MOSAIC under different ablation conditions on the HDTesting dataset, evaluated using AUC, AUPR, and MCC. (a) Module-level ablation: removing the multi-scale convolution module or transformer encoder module, and replacing the gated fusion module with simple additive fusion. (b) Feature-level ablation: removing DNA2vec, GPN-MSA, or functional annotation features.

Ablation analysis was used to assess the contributions of MOSAIC features and model components. Among the three feature categories, GPN-MSA features contributed most to model performance (Fig. 5a and Supplementary Table S12), underscoring the importance of evolutionary and functional context [31]. Functional annotation features also played a major role by providing biologically relevant regulatory information [17, 24], while DNA2vec features offered complementary local sequence patterns. At the model level, the transformer encoder had the greatest effect on performance, with its removal decreasing the AUC from 96.1% to 92.8% (Fig. 5b and Supplementary Table S13). The multi-scale convolution and gated fusion modules also contributed to performance, as ablation of either module led to measurable declines. Together, these results show that MOSAIC achieves strong performance through the joint contribution of complementary input features and core architectural modules.

### Interpretability of the MOSAIC model

#### Interpretability of features

**Figure 6:**
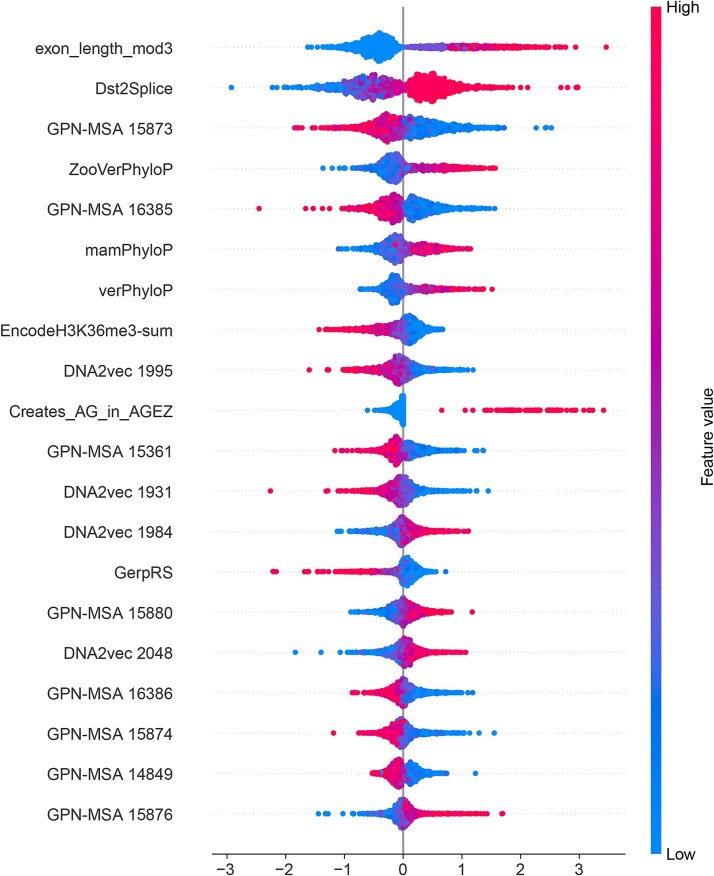
SHAP feature importance analysis of MOSAIC.

SHAP analysis of MOSAIC outputs revealed distinct contributions from each feature category to model predictions (Fig. 6). Functional annotation features ranked highest, indicating that prior biological knowledge of noncanonical SAVs provided critical discriminative information for the model. GPN-MSA features also contributed substantially, underscoring their importance in capturing long-range semantic relationships and evolutionary constraints. In addition, DNA2vec features exhibited ranked moderately in importance, reflecting their complementary role in providing local sequence context around variants. Overall, these findings suggest that MOSAIC primarily relies on high-level functional annotation features and GPN-MSA features when discriminating the pathogenicity of noncanonical SAVs, whereas local sequence fragment features serve a secondary yet complementary role.

#### Visualization of discriminative representations for noncanonical SAVs

**Figure 7:**
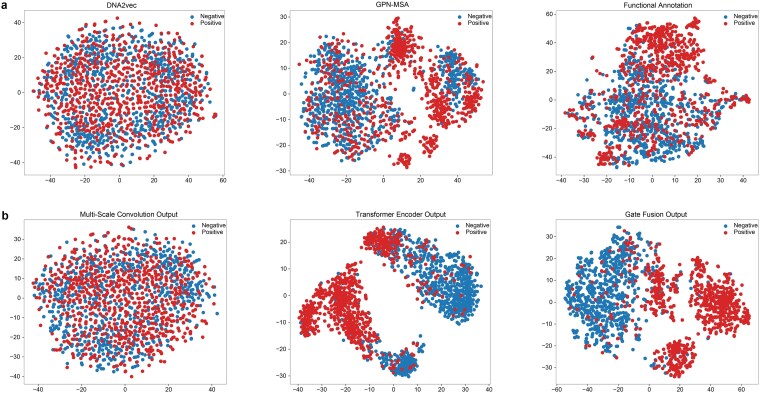
Visualization of learned representation. Two-dimensional t-SNE projections illustrate the distributions of representations at different stages within MOSAIC. (a) Original input features, including DNA2vec, GPN-MSA, and functional annotation features. (b) Learned representations at key stages within the MOSAIC model.

The t-SNE visualization of the original input features—DNA2vec, GPN-MSA, and functional annotation features—showed poor separation between pathogenic and benign variants (Fig. 7a). DNA2vec features were highly mixed, GPN-MSA features were diffusely distributed, and functional annotation features, although slightly more structured, still showed substantial overlap. In contrast, intermediate and final representations learned by MOSAIC displayed progressively improved separation. After the multi-scale convolution module, DNA2vec features became more compact, and subsequent transformer encoding further enhanced cluster formation. The final fused representation showed the clearest class separation, with greater inter-class distance and tighter intra-class clustering (Fig. 7b). To further illustrate how MOSAIC partitions the learned representation space, we visualized an approximate decision boundary based on the fused features from the Gate Fusion layer (Supplementary Fig. S3). Collectively, these results indicate that MOSAIC progressively transforms raw, poorly separable features into highly discriminative representations, enabling accurate classification of noncanonical SAVs.

#### DNA sequence motif analysis

**Figure 8:**
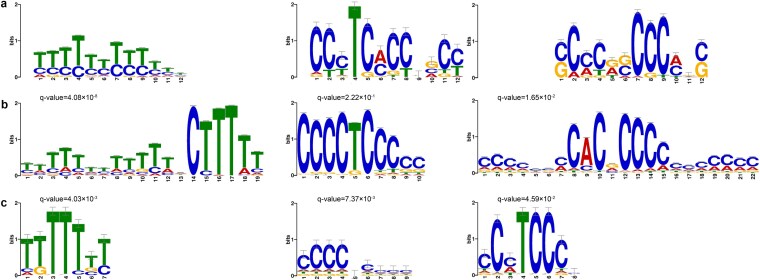
DNA sequence motif analysis. High-weight candidate motifs identified from the MOSAIC attention pooling layer are shown together with their best-matching motifs from external databases. (a) Three high-weight candidate motifs learned by MOSAIC. (b) Best-matching transcription factor motifs from the JASPAR database. (c) Best-matching RNA-binding protein motifs from the CISBP-RNA database. The significance of each match is indicated by the corresponding q-value.

To investigate the sequence features emphasized by MOSAIC, the top 10 candidate motifs with the highest significance selected from the MOSAIC attention pooling layer were compared to evaluate their similarity to known functional motifs. Comparison of these motifs with experimentally validated transcription factor DNA-binding motifs from the JASPAR database [43] revealed that three candidate motifs corresponded to known sites (Fig. 8a), with two reaching statistical significance (q = 4.08 × 10^−8^ and q = 1.65 × 10^−2^) and one not reaching statistical significance (q = 2.22 × 10^−1^) (Fig. 8b). The same three candidate motifs also matched human RBP binding motifs in the CISBP-RNA database [44], with q-values of 4.03 × 10^−3^, 7.37 × 10^−3^, and 4.59 × 10^−2^, respectively (Fig. 8c). These results indicate that MOSAIC assigns high-weight attention to sequence elements overlapping known transcription and RBP binding motifs. The findings also suggest that the corresponding noncanonical SAVs prioritized by the model may disrupt conserved regulatory elements (including TFs and RBPs), revealing a potential mechanism for their pathogenicity.

### Pathogenic noncanonical SAVs are preferentially located in active regulatory regions and RBP binding sites

**Figure 9:**
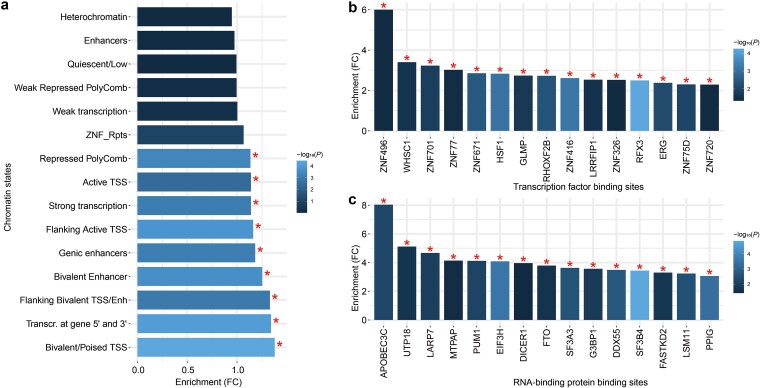
Regulatory feature enrichment of pathogenic noncanonical SAVs. (A) Chromatin states. (B) TF-binding sites. (C) RBP-binding sites. Bar heights indicate the observed fold enrichment of pathogenic noncanonical SAVs in each regulatory feature relative to random background variants. Bar colors represent the statistical significance measured by −log₁₀(P). Red asterisks indicate statistically significant results (*P* < .05). For TF and RBP binding sites, only the top 15 annotations ranked by fold enrichment are displayed.

Accumulating evidence shows that SAVs are enriched among various genetic regulatory elements [46–50]. Therefore, we further evaluated functional enrichment patterns of pathogenic noncanonical SAVs predicted by MOSAIC among genetic regulatory elements (i.e. chromatin states, TF, and RBP binding sites). We observed that these predicted pathogenic noncanonical SAVs were significantly enriched in actively transcribed regions and regulatory regions, but not in heterochromatic or transcriptionally silent regions (Fig. 9a). Significant enrichment was also observed in binding sites of 575 TFs, including ZNF496, WHSC1, and HSF1 (Fig. 9b and Supplementary Table S14). We also found the significant enrichment of noncanonical SAVs in the binding sites of 38 RBPs, including APOBEC3C, UTP18, LARP7, FTO, and PUM1 (Fig. 9c and Supplementary Table S15). The strongest enrichment of variants occurred in RBP binding sites across the three regulatory feature types, aligning with prior evidence linking SAVs pathogenicity to RBP binding [51, 52]. Collectively, these findings confirm that MOSAIC-predicted pathogenic noncanonical SAVs are significantly enriched in active regulatory regions, TF binding sites, and RBP binding sites, relative to control variants, offering mechanistic support for the reliability of model predictions.

## Discussion

Accurately predicting the pathogenic potential of noncanonical SAVs remains a significant challenge in functional genetic variant interpretation. Unlike canonical SAVs that directly disrupt consensus splicing signals, noncanonical SAVs often perturb splicing by affecting the complex splicing regulatory environment. Their pathogenic mechanisms are more elusive and heterogeneous, which substantially increases the difficulty of prediction. To address this challenge, we present MOSAIC, a computational framework for predicting the pathogenicity of noncanonical SAVs. Within a unified modeling architecture, MOSAIC integrates local sequence representations, long-range contextual dependencies, and functional annotations associated with noncanonical SAVs. This integration enables the model to comprehensively capture the complex regulatory patterns of noncanonical SAVs from multiple perspectives. Compared to existing methods, MOSAIC demonstrates particularly strong performance in the task of noncanonical SAV prediction.

The superior performance of MOSAIC likely stems from its multimodal feature integration strategy and its ability to capture both local and long-range regulatory signals, consistent with previous studies showing the value of integrating diverse biological features and advanced modeling frameworks in genomic prediction tasks [53–55]. Further discussion is provided in the Supplementary Discussion.

In clinical applications, one of the key challenges for pathogenicity prediction lies in the fact that neutral variants significantly outnumber pathogenic variants. MOSAIC effectively addresses this issue and exhibits outstanding performance in predicting pathogenicity for rare noncanonical SAVs. On the VarRareTest dataset, MOSAIC achieved an AUC of 0.954, markedly outperforming current tools (Fig. 4a). Notably, although DNA2vec has known limitations in representing rare sequence patterns and does not explicitly enforce reverse-complement invariance, the strong performance on this independent test set, together with the multimodal design of MOSAIC, suggests that these limitations do not materially affect overall predictive performance. In addition, sequence motifs assigned high predictive importance by MOSAIC show strong consistency with experimentally validated TFs and RBPs. This consistency offers direct molecular evidence supporting the pathogenicity of high-risk noncanonical SAVs predicted by the model and substantially enhances the biological credibility of the predictions.

To evaluate the contribution of epigenetic features, we conducted an ablation analysis on the HDTesting dataset. Removing epigenetic features from the full model led to a consistent decrease across all major metrics (Supplementary Table S16), including AUC (0.961 to 0.956), AUPR (0.968 to 0.965), and F1 score (0.910 to 0.899). These results suggest that although epigenetic annotations may lack tissue-specificity, they provide complementary regulatory information that contributes to the overall predictive performance of MOSAIC.

Although MOSAIC showed strong predictive performance, several limitations remain, including dependence on curated germline variant databases, incomplete representation of unannotated pathogenic mechanisms, and the need for experimental validation. More detailed discussion is provided in the Supplementary Discussion. Additionally, the observed performance differences may partly reflect differences in training data and labeling strategies rather than model architecture alone. MOSAIC was trained using clinically curated variant databases such as ClinVar and HGMD, whereas other methods rely on diverse data sources, including reference annotations, functional genomics signals, or large-scale unsupervised training. These differences in data construction may also contribute to performance variations across methods.

## Conclusion

This study introduced MOSAIC, a deep learning framework based on multimodal feature fusion for predicting the pathogenicity of noncanonical SAVs. By simultaneously capturing long-range contextual dependencies, local sequence patterns, and splicing-associated functional annotations, MOSAIC achieved consistently high predictive performance across diverse benchmark datasets. Interpretability analyses further demonstrated that the high-weight sequence patterns captured by the model closely aligned with known TF and RBP motifs, providing direct mechanistic insights into the regulatory disruptions caused by pathogenic variants. Together, these results establish MOSAIC as a robust and interpretable tool for noncanonical SAV pathogenicity prediction, offering valuable computational support for genetic disease research and precision medicine.

**Key Points**

- Noncanonical splice-altering variants (SAVs) present a significant challenge in clinical interpretation due to complex regulatory mechanisms and data scarcity.
- We present MOSAIC, deep learning framework designed to predict the pathogenicity of noncanonical SAVs by integrating long-range contextual signals derived from a pretrained DNA language model, local sequence features captured from multi-scale convolutional neural networks, and functional annotations.
- Extensive benchmarking on independent datasets demonstrates that MOSAIC achieves superior performance compared to state-of-the-art tools, facilitating more accurate genetic diagnosis.
- By leveraging SHAP values and motif analysis, MOSAIC provides interpretability for its predictions, revealing critical *cis*-regulatory elements and offering biological insights into splicing mechanisms.

## Supplementary Material

Supplementary_Materials_20260507_bbag291

## Data Availability

All public data and tools used in this study are referenced in the main text or the Supplementary Materials. Pathogenic splice-altering variants from HGMD Professional were obtained from the HGMD website (http://www.hgmd.cf.ac.uk/) under a commercial license. Due to HGMD licensing restrictions, these variants cannot be shared publicly. Benign splice-altering variants from ClinVar are publicly available from the NCBI ClinVar database (https://ftp.ncbi.nlm.nih.gov/pub/clinvar/). Whole-genome SNV data provided by CADD are publicly available from the CADD download page (https://cadd.gs.washington.edu/download). Chromatin state annotations from the Roadmap Epigenomics Project can be downloaded from its data portal (https://egg2.wustl.edu/roadmap/web_portal/processed_data). Transcription factor ChIP-seq peak files are available from the ENCODE database (https://www.encodeproject.org/). RBP-related data from CLIPdb can be downloaded from http://clipdb.ncrnalab.org. Data generated in this study are available from the corresponding author upon reasonable request.
