# Supplementary material for: Pathogenicity prediction for noncanonical splice-altering variants based on multimodal feature fusion
Source: Brief Bioinform. 2026 Jun 4;27(3):bbag291. doi: 10.1093/bib/bbag291 (PMC13365626; doi:10.1093/bib/bbag291)
Supplement: Supplementary_Materials_20260507_bbag291 [file supplementary_materials_20260507_bbag291.zip › Supplementary Materials 20260507.docx]

**Supplementary Materials**

**Supplementary Methods**

**Data Preparation**

The dataset integrated multiple variant databases based on the GRCh38/hg38 human genome assembly, with variants restricted to single-nucleotide variants (SNVs). Pathogenic variants were obtained from HGMD Professional (version 2023.03)[1], restricted to those labeled as “DM” (disease-causing mutation) and annotated as splice or exonic-splice variants. A single variant located on the Y chromosome was excluded due to insufficient sample representation. Only variants located on chromosomes 1 through 22 and chromosome X were retained. Benign variants were collected from the ClinVar[2] database using the VCF file released on 1 December 2024. Variants annotated as “benign,” “likely benign,” or “benign/likely benign” were selected, and their review status was required to meet one of the following criteria: “criteria provided, multiple submitters, no conflicts,” “criteria provided, single submitter,” or “reviewed by expert panel.” To focus on non-canonical SAVs, the filtered benign variants were annotated using Ensembl Variant Effect Predictor (VEP, release 113)[3]. We retained only those assigned to specific non-canonical splice categories, including splice region variant, splice donor region variant, splice donor 5th base variant, and splice polypyrimidine tract variant. Variants with conflicting interpretations or unknown clinical significance were excluded. After filtering, 4,705 pathogenic variants and 22,868 benign variants were obtained. To address class imbalance, a genome position-based close-by strategy[4] was used to construct a balanced dataset. Specifically, for each pathogenic variant, the benign variant located closest in genomic position on the same chromosome was selected as the matched negative control, without imposing a fixed distance threshold. This nearest-neighbor matching strategy was adopted to reduce potential confounding from local genomic context while avoiding arbitrary distance cutoffs. Pathogenic variants without a suitable matched benign variant were excluded, yielding a final balanced pool of 3,873 pathogenic and 3,873 benign variants.

To evaluate the generalization ability of the model, the constructed dataset was partitioned into a training set and an independent test set, designated as HDTesting, using a 4:1 split ratio. Within the training set, 20% of the data were randomly sampled as a validation set for hyperparameter tuning and model selection. A secondary test set, HDSTesting, was assembled from HGMD variants excluded during training due to the absence of suitable nearby benign counterparts. This dataset contained 832 pathogenic variants, and an equal number of benign variants were randomly selected from the remaining unused benign pool to preserve a balanced 1:1 class ratio. The resulting HDSTesting dataset comprised 1,664 variants.

In addition, a rare variant test set, VarRareTest, was constructed by extracting variants with an allele frequency below 0.01 from the HDTesting dataset. Allele frequency data were obtained from the gnomAD database (version 4.1)[5]. Variants lacking allele frequency information were excluded. After filtering, the VarRareTest dataset included 515 rare variants, consisting of 173 pathogenic and 342 benign variants.

Gene-level features capture attributes intrinsic to genes and are therefore shared by all variants mapped to the same gene. The use of such features may cause all variants within a gene to assign identical feature values, potentially introducing bias and increasing the risk of label leakage. To mitigate this effect, variants originating from genes represented in the HGMD-Training dataset were excluded from the HDTesting dataset, ensuring complete gene-level separation between training and evaluation data. After this filtering step, the resulting HDGTesting dataset contained 159 variants, including 57 pathogenic and 102 benign variants.

**Functional annotation features construction**

***Splicing-specific Features***

Splicing-specific features were used to assess the potential impact of variants on the splicing signals and surrounding cis-regulatory elements. Utilizing genome-wide annotations from CADD (version 1.7), we extracted for each variant its distance (with direction) to the nearest donor and acceptor splice sites. Based on these distances, variants were classified as residing within the splice-site core (0–2 nt) or extended (2–6 nt) regions, which are known to be critical for accurate splice-site recognition and are therefore particularly sensitive to sequence perturbations[6–8]. In addition, we examined whether variants introduce novel AG or YAG motifs within the AG exclusion region upstream of the 3’ splice sites (defined as −50 to −3 nt on the intronic side). Motif introduction in this region can disrupt the polypyrimidine tract and promote activation of cryptic acceptor sites. Therefore, this feature was used to identify variants with potential cryptic splice acceptor risk[7].

***Sequence and Genomic Structure Features***

Sequence and structural features were incorporated to characterize the local genomic context surrounding each variant. The GC content within a central window of each variant was derived from the precomputed genome-wide annotations provided by CADD. This feature reflects the potential impact of local DNA structure, nucleosome occupancy, and transcriptional activity on splicing outcomes. CpG island annotations were also obtained to determine whether variants located within CpG-rich regulatory regions. In addition, distances from each variant to the nearest transcription start site and transcription termination site were extracted, as these distances may indicate whether variants are located within key regulatory regions. Furthermore, we calculated the length of the exon harboring the variant and its modulo three (mod3). The mod3 value was used to determine whether the variant's exon maintains the reading frame structure, as reading frame integrity is closely associated with the tolerance of alternative splicing[9].

***Conservation Scores***

Conservation scores were used to evaluate evolutionary constraint at variant loci across multiple species. Based on the precomputed genome-wide annotations from CADD, we extracted cross-species conservation features. These include PhyloP and PhastCons scores sourced from UCSC, which span varying evolutionary depths such as primates, placental mammals, and vertebrates, providing single-nucleotide-resolution conservation metrics. To further characterize evolutionary constraint, GERP-derived scores (including GerpN and GerpS) were included to identify regions under strong negative selection. All conservation metrics were derived from multi-species whole-genome alignments. For variants with missing PhyloP/PhastCons scores, the mean score of the corresponding species clade was used for imputation. Undefined GERP constraint scores were uniformly set to zero[6].

***Epigenetic Features***

Epigenetic features were included to capture the influence of chromatin states on gene expression and splicing regulation. Previous studies have shown that specific histone modifications, chromatin accessibility, and transcriptional activity levels can significantly influence exon recognition and alternative splicing by recruiting splicing factors or modulating RNA polymerase II elongation rates[10,11]. Therefore, we extracted epigenetics-related features from the CADD annotations, which aggregate signals across multiple biological contexts and serve as non-tissue-specific functional annotations. These features, sourced from the ENCODE[12] and Roadmap Epigenomics projects[13], encompass signals from various histone modifications (H3K4me1, H3K4me2, H3K4me3, H3K9ac, H3K9me3, H3K27ac, H3K27me3, H3K36me3, H3K79me2, H4K20me1, H2AFZ), chromatin accessibility (DNase I hypersensitivity), and transcriptional activity. Both the regional cumulative value (sum) and the local peak signal (max) for each feature were used as model inputs[6]. For each splice-altering variant, the corresponding epigenetic annotations were extracted from CADD and combined with the variant-related features as model inputs, followed by integration through the feature fusion module.

**Regulatory Feature Enrichment Analysis of Non-canonical SAVs**

Accumulating evidence indicates that splicing regulation involves not only RNA splicing factors but also transcription factors and chromatin state dynamics[14–17]. To evaluate the enrichment patterns of pathogenic non-canonical SAVs across diverse regulatory features, we performed regulatory enrichment analysis. Initially, human genome-wide single-nucleotide variant data from CADD[6] were annotated using VEP[3]. Variants assigned one of the annotations “splice_donor_5th_base_variant”, “splice_region_variant”, “splice_donor_region_variant”, or “splice_polypyrimidine_tract_variant” were retained. This yielded a genome-wide candidate set of 10,902 non-canonical SAVs. These variants were subsequently scored using MOSAIC, which assigned pathogenicity probabilities ranging from 0 to 1. Variants with scores exceeding 0.5 were classified as pathogenic, resulting in a subset of 7,691 predicted pathogenic non-canonical SAVs used for downstream enrichment analysis.

To investigate the functional enrichment of these predicted pathogenic non-canonical SAVs in various regulatory elements, we downloaded relevant genomic regulatory annotation data. Chromatin state annotations were derived from BED files generated by the 15-state ChromHMM model from the Roadmap Epigenomics Project[13]. This model integrates five core histone marks (H3K4me3, H3K4me1, H3K36me3, H3K27me3, and H3K9me3) to segment the genome into 15 functional states, which are used to characterize various types of regulatory functional regions, including promoters, enhancers, transcriptional elongation regions, and repressed regions. Next, TF-binding data were obtained from the ENCODE database[12]. ChIP-seq peak files in BED format were batch-downloaded from the ENCODE portal, restricted to *Homo sapiens* samples and filtered for datasets labeled as “released”. After unified quality control and deduplication, high-confidence binding site sets for 1,147 distinct TFs were obtained and used as reference annotations for subsequent enrichment analysis. Furthermore, pre-mRNA splicing is regulated by a protein-RNA interaction network that consists of cis-regulatory elements and trans-acting factors (primarily RBPs)[18,19]. To investigate whether pathogenic non-canonical SAVs predicted by MOSAIC are enriched within RBP binding sites, CLIP-seq binding peaks in BED format were retrieved for 220 human RBPs from the CLIPdb database[20]. These data were used to construct a high-confidence RBP binding landscape for enrichment analysis.

Regulatory feature enrichment analysis was performed on the 7,691 pathogenic non-canonical SAVs predicted by MOSAIC using GREGOR (Genomic Regulatory Elements and GWAS Overlap Algorithm)[21]. GREGOR evaluates enrichment by accounting not only for the index variants but also for proxy variants in linkage disequilibrium (LD, r² cutoff = 0.7). In parallel, a background set of control variants was randomly sampled from the genome and matched to the candidate set in terms of allele frequency, gene proximity, and LD structure. These matched variants served as a baseline for comparison. The enrichment significance of candidate variants across different regulatory features was then assessed relative to these matched controls. **Supplementary Discussion**

**Possible reasons for the superior performance of MOSAIC**

The superior predictive performance of MOSAIC primarily arises from two key design features. First, the model adopts a multi-modal feature fusion strategy that integrates DNA2vec-derived embeddings, GPN-MSA evolutionary constraint signals, and functional annotations related to non-canonical SAVs within a shared representation space. This integration enables MOSAIC to jointly model distinct but complementary regulatory signals, capturing global sequence composition, evolutionary conservation, and functional relevance, thereby providing a more comprehensive feature basis for pathogenicity discrimination. Second, the architectural framework of MOSAIC is optimized to capture both local and distal determinants of splicing regulation. The multi-scale convolutional module extracts local sequence features across different length scales, while the transformer encoder models long-range contextual dependencies via self-attention. In addition, the gated fusion mechanism further refines the contribution of each feature class, adaptively balancing their influence to prevent overfitting to any single modality and allowing robust learning of integrated regulatory effects underlying non-canonical splicing events. Similar computational frameworks that integrate diverse biological features and advanced modeling strategies have also demonstrated strong performance in variant pathogenicity prediction and related genomic prediction tasks[22–24].

**Limitations and future directions**

Despite its strong performance and broad application potential, certain limitations remain. First, model training currently relies on manually curated variant databases such as ClinVar[2] and HGMD[1]. Although these resources provide high-quality labels, their coverage remains limited. In addition, these databases primarily catalog germline variants, potentially limiting the representativeness of the training data for somatic mutational contexts, particularly in oncology. Consequently, the model may be subject to distribution shift when applied to somatic variants, and its predictions in such settings should be interpreted with caution. For novel variants that are not yet thoroughly annotated or lack sufficient supporting evidence, the model's generalization capability still needs further improvement. Second, MOSAIC incorporates 47 pre-defined biological features spanning splicing regulation, evolutionary conservation, and chromatin state. Although these features effectively capture known regulatory mechanisms, they may overlook unannotated or novel pathogenic mechanisms due to reliance on expert curation. Future development will focus on incorporating deep learning approaches that place greater emphasis on automatic feature learning. Such approaches may reduce dependence on manual annotations and further enhance the model’s ability to comprehensively characterize the pathogenic mechanisms of non-canonical splicing variants. Finally, current predictions are supported primarily by computational inference and statistical enrichment derived from public datasets. Experimental validation remains essential for clinical translation. Future work will integrate functional assays such as *in* *vitro* minigene splicing and single-cell long-read sequencing to systematically evaluate the predicted high-risk variants and elucidate their mechanistic consequences.

**Supplementary Figure**


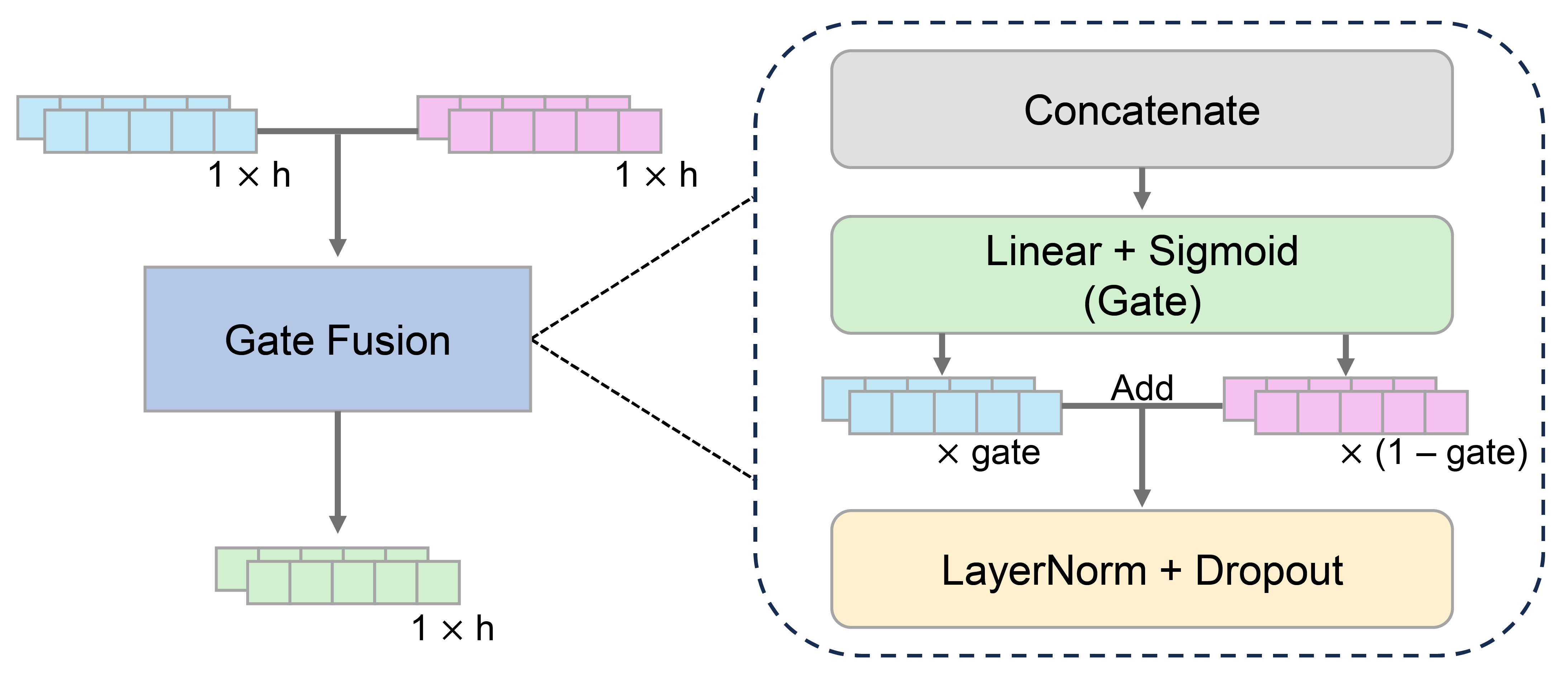


**Supplementary Figure S1. Gated fusion module.** Sequence representations and functional annotation features are adaptively fused via a learnable gating vector. Feature-wise weights are generated through a fully connected layer with Sigmoid activation and applied element-wise to form a unified representation.


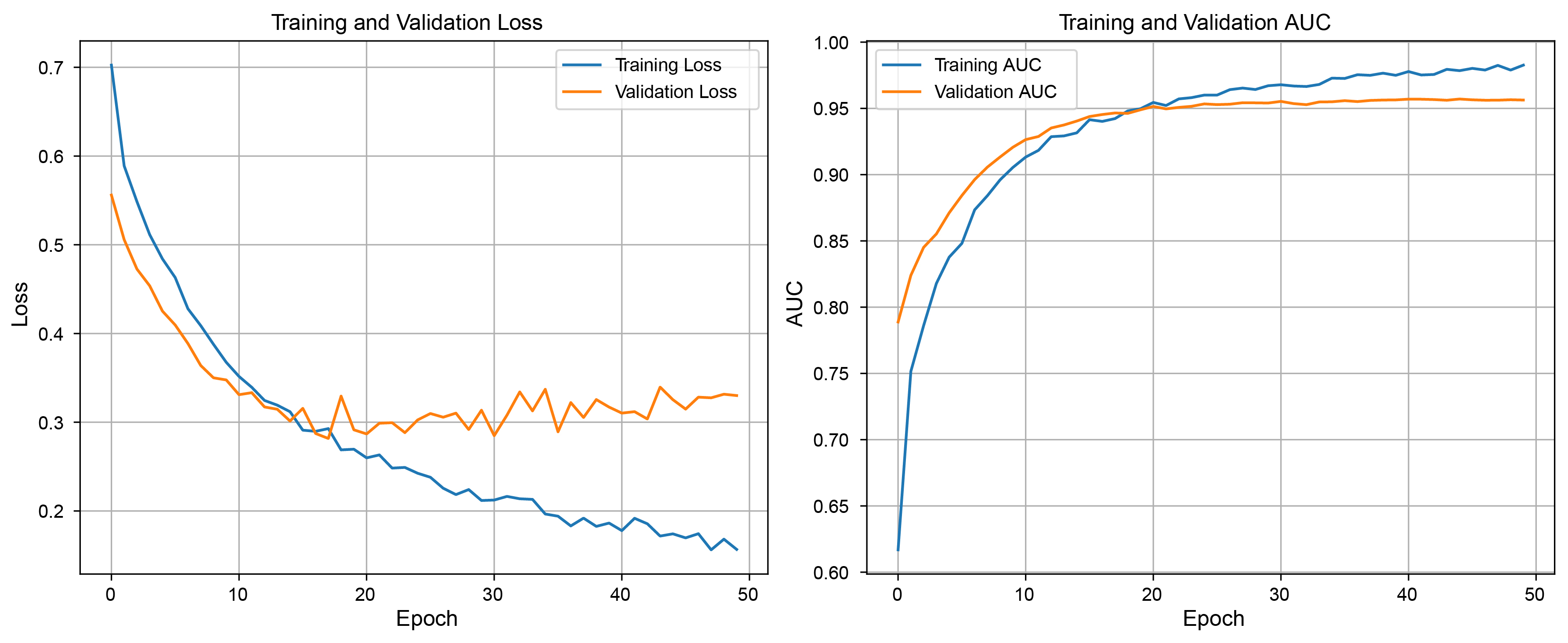


**Supplementary Figure S2. Training and validation performance curves.** The training loss, validation loss, training AUC, and validation AUC are shown across epochs during model optimization. Loss curves illustrate the convergence behavior of the model, while AUC curves reflect the predictive performance on both the training and validation sets. The best-performing model was selected according to the highest validation AUC.


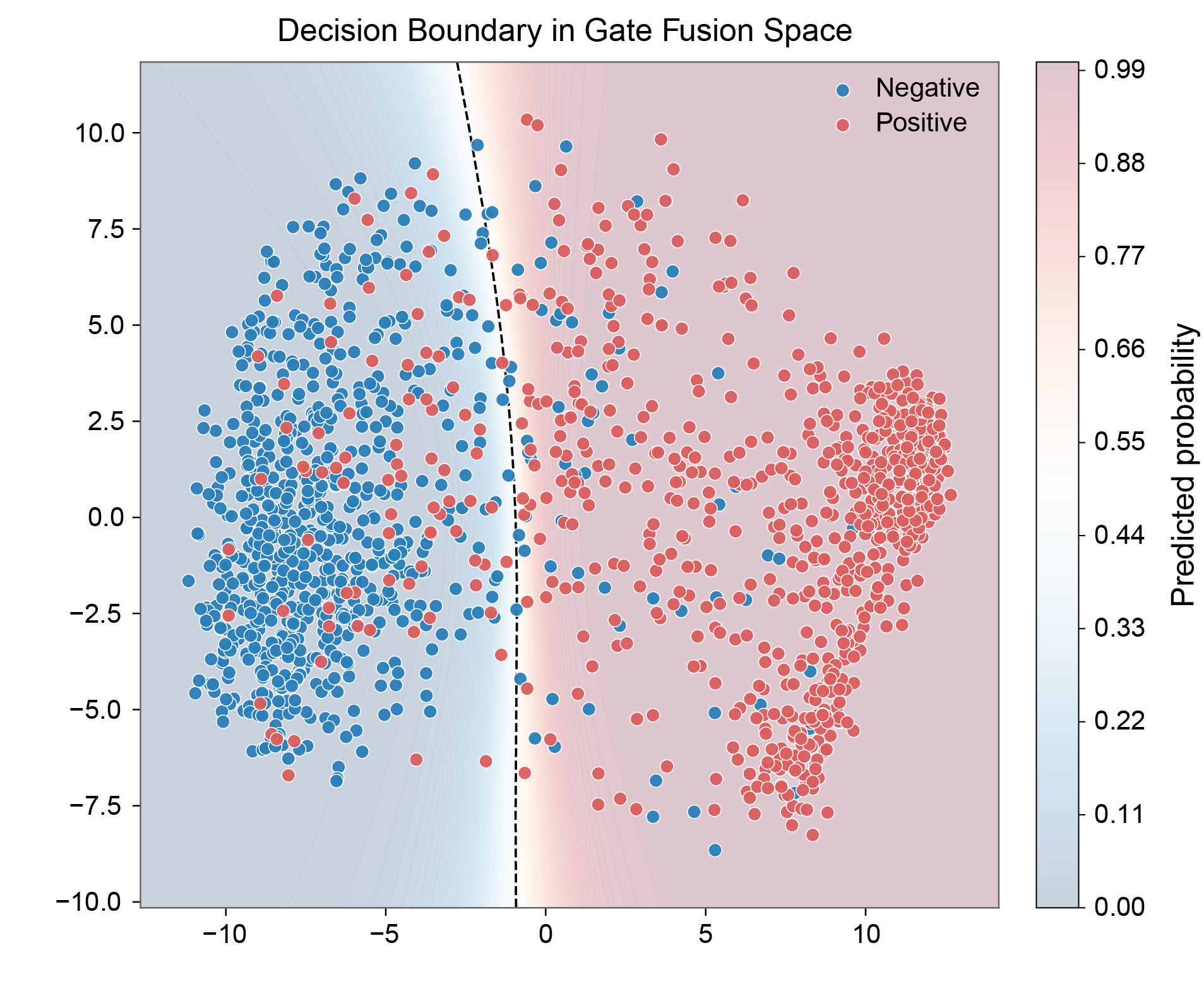


**Supplementary Figure S3. Approximate decision boundary visualization in the Gate Fusion feature space of MOSAIC.** Fused feature representations were projected onto a two-dimensional plane using principal component analysis (PCA). A dense grid was generated in the projected space and approximately mapped back to the original high-dimensional feature space via inverse transformation. Each grid point was then evaluated by the final classification layer to obtain the predicted probability of the positive class. The background color indicates the predicted probability (blue to red), and the black contour marks the decision boundary at a probability threshold of 0.5. Overlaid data points show the distribution of negative and positive samples relative to the decision regions, illustrating how MOSAIC partitions the fused feature space.

**References**

1. Stenson PD, Mort M, Ball EV *et al.* The Human Gene Mutation Database (HGMD®): optimizing its use in a clinical diagnostic or research setting. *Hum Genet* 2020;**139**(10):1197–207.

2. Landrum MJ, Chitipiralla S, Brown GR *et al.* ClinVar: improvements to accessing data. *Nucleic Acids Res* 2020;**48**(D1):D835–44.

3. McLaren W, Gil L, Hunt SE *et al.* The Ensembl Variant Effect Predictor. *Genome Biol* 2016;**17**(1):122.

4. Ritchie GRS, Dunham I, Zeggini E *et al.* Functional annotation of noncoding sequence variants. *Nat Methods* 2014;**11**(3):294–6.

5. Karczewski KJ, Francioli LC, Tiao G *et al.* The mutational constraint spectrum quantified from variation in 141,456 humans. *Nature* 2020;**581**(7809):434–43.

6. Schubach M, Maass T, Nazaretyan L *et al.* CADD v1.7: using protein language models, regulatory CNNs and other nucleotide-level scores to improve genome-wide variant predictions. *Nucleic Acids Res* 2024;**52**(D1):D1143–54.

7. Danis D, Jacobsen JOB, Carmody LC *et al.* Interpretable prioritization of splice variants in diagnostic next-generation sequencing. *Am J Hum Genet* 2021;**108**(11):2205.

8. Cheng J, Nguyen TYD, Cygan KJ *et al.* MMSplice: modular modeling improves the predictions of genetic variant effects on splicing. *Genome Biol* 2019;**20**(1):48.

9. Lord J, Gallone G, Short PJ *et al.* Pathogenicity and selective constraint on variation near splice sites. *Genome Res* 2019;**29**(2):159–70.

10. Luco RF, Pan Q, Tominaga K *et al.* Regulation of alternative splicing by histone modifications. *Science* 2010;**327**(5968):996–1000.

11. Kornblihtt AR. Chromatin, transcript elongation and alternative splicing. *Nature Structural & Molecular Biology* 2006;**13**(1):5–7.

12. ENCODE Project Consortium, Moore JE, Purcaro MJ *et al.* Expanded encyclopaedias of DNA elements in the human and mouse genomes. *Nature* 2020;**583**(7818):699–710.

13. Roadmap Epigenomics Consortium, Kundaje A, Meuleman W *et al.* Integrative analysis of 111 reference human epigenomes. *Nature* 2015;**518**(7539):317–30.

14. Van Nostrand EL, Freese P, Pratt GA *et al.* A large-scale binding and functional map of human RNA-binding proteins. *Nature* 2020;**583**(7818):711–9.

15. Tian J, Chen C, Rao M *et al.* Aberrant RNA Splicing Is a Primary Link between Genetic Variation and Pancreatic Cancer Risk. *Cancer Res* 2022;**82**(11):2084–96.

16. GTEx Consortium. The GTEx Consortium atlas of genetic regulatory effects across human tissues. *Science* (New York, N.Y.) 2020;**369**(6509):1318–30.

17. Walker RL, Ramaswami G, Hartl C *et al.* Genetic Control of Expression and Splicing in Developing Human Brain Informs Disease Mechanisms. *Cell* 2019;**179**(3):750-771.e22.

18. Park E, Pan Z, Zhang Z *et al.* The Expanding Landscape of Alternative Splicing Variation in Human Populations. *Am J Hum Genet* 2018;**102**(1):11–26.

19. Wang Z, Burge CB. Splicing regulation: from a parts list of regulatory elements to an integrated splicing code. *RNA* (New York, N.Y.) 2008;**14**(5):802–13.

20. Yang YCT, Di C, Hu B *et al.* CLIPdb: a CLIP-seq database for protein-RNA interactions. *BMC Genomics* 2015;**16**(1):51.

21. Schmidt EM, Zhang J, Zhou W *et al.* GREGOR: evaluating global enrichment of trait-associated variants in epigenomic features using a systematic, data-driven approach. *Bioinformatics* (Oxford, England) 2015;**31**(16):2601–6.

22. Yue Z, Chu X, Xia J. PredCID: prediction of driver frameshift indels in human cancer. *Brief Bioinform* 2021;**22**(3):bbaa119.

23. Xiang Y, Li X, Gao Q *et al.* ExplainMIX: Explaining Drug Response Prediction in Directed Graph Neural Networks With Multi-Omics Fusion. *IEEE J Biomed Health Inform* 2025;**29**(7):5339–49.

24. Gao Q, Xu T, Li X *et al.* Interpretable Dynamic Directed Graph Convolutional Network for Multi-Relational Prediction of Missense Mutation and Drug Response. *IEEE J Biomed Health Inform* 2025;**29**(2):1514–24.
